# Inhibition of USP14 suppresses the formation of foam cell by promoting CD36 degradation

**DOI:** 10.1111/jcmm.15002

**Published:** 2020-01-22

**Authors:** Fangcheng Zhang, Xiaohong Xia, Renjie Chai, Ruqin Xu, Qiong Xu, Mingke Liu, Xuke Chen, Bin Liu, Shiming Liu, Ningning Liu

**Affiliations:** ^1^ Guangzhou Institute of Cardiovascular Disease Guangdong Key Laboratory of Vascular Diseases State Key Laboratory of Respiratory Disease the Second Affiliated Hospital Guangzhou Medical University Guangzhou China

**Keywords:** Atherosclerosis, CD36, degradation, foam cell, USP14

## Abstract

Atherosclerosis is regarded as a chronic progressive inflammatory disease and is a basic pathophysiological process in coronary artery disease which is life threatening in clinic. The formation of foam cell plays a key role in the pathogenesis of atherosclerosis. OxLDL is a significant factor in progression of coronary artery disease. Our studies have demonstrated that USP14 promotes cancer development and mediates progression of cardiac hypertrophy and LPS‐induced inflammation. However, the underlying mechanism of USP14 is unknown. In this study, we found that the inhibition of USP14 significantly suppressed the oxLDL uptake, subsequently decreased the foam cell formation. Surprisingly, USP14 has an effect on the expression of CD36 but not SR‐A, ABCA1, Lox‐1, ABCG1 and SR‐Bl. Furthermore, USP14 stabilizes CD36 protein via cleaving the ubiquitin chain on CD36. Blocking CD36 activation using antibody‐dependent blocking assay remarkably attenuated the function of USP14 on the formation of foam cell. In summary, our results suggested that the inhibition of USP14 decreases foam cell formation by down‐regulating CD36‐mediated lipid uptake and provides a potential therapeutic target for atherosclerosis.

## 1 INTRODUCTION

Atherosclerosis (AS) is the most common disease worldwide and remains to be the leading cause of mortality, including coronary heart disease, peripheral artery and stoke.^1^, ^2^, ^3^ It is well known that atherosclerosis, a basic pathophysiological artery disease, is a chronic and progressive inflammatory reaction process.^4^ The occurrence of myocardial infarction, sudden death or stroke results from atherosclerotic plaque rupture and thrombosis. At present, it has been reported that foam cell formation is a key link in early stage of atherosclerosis.^5^ In pathological conditions such as hypertension and hyperlipidemia, low‐density lipoprotein (LDL) is deposited under the subendothelial space and oxidized into oxLDL. Stimulated by the strong inflammatory factor, monocytes are activated into the endodermis and become macrophages. Macrophages intake modified low‐density lipoproteins, including oxidized‐LDL (oxLDL) and acetylated‐LDL (acLDL), resulting in the accumulation of large amounts of cholesterol lipids and become foam cell through scavenger receptors, such as CD36, lectin‐like oxLDL receptor‐1 (Lox‐1) and scavenger receptor A type 1 (SR‐A).^6^, ^7^ ATP binding cassette transporters A1, G1 (ABCA1 and ABCG1) and SR‐B1 mediate cholesterol efflux.^8^, ^9^, ^10^ Cellular lipid uptake and inhibition of intracellular lipid efflux are core in the transformation from macrophages to foam cell.

CD36 is located in the pit of macrophage cell membrane and is a key member of scavenger receptor family.^11^ CD36, considered as a receptor for oxLDL, has a vital position in foam cell formation.^12^, ^13^, ^14^ The uptake of oxLDL by macrophages is decreased by blocking CD36 by 50%.^15^ The capacity of monocytes for oxLDL binding lacking functional CD36 expression remarkably is weakened. In addition, foam cell formation and the development of atherosclerosis are inhibited in animal model of CD36 deletion.^16^ On the other hand, oxLDL induces the up‐regulation of macrophage CD36 in the level of protein and gene, indicating a positive feedback loop in the cause of foam cell formation.^17^ Thus, further exploring the mechanisms of CD36 expression will be significant for a novel therapeutic scheme for atherosclerosis.

Ubiquitin‐proteasome system (UPS) regulates the most of protein degradation. 26S proteasome, consisting of a 19S regulatory particle and a 20S proteasome in the UPS, plays a important role in promoting targeted protein degradation. In the process of protein degradation, deubiquitinating enzymes (DUBs) are essential, removing ubiquitin/ubiquitin‐like (Ub/Ubl) chains. In the UPS exits approximately 100 putative DUBs in human genome.^18^ In the 19S regulatory particle in mammalian, there are three DUBs, including UCHL5, Rpn11 and USP14.^19^, ^20^ USP14 can reduce the anchoring time of ubiquitin conjugates and stabilize the proteins via inducing deubiquitination of proteins.^21^ Moreover, in our previous study, we have reported that USP14 was involved in inflammatory diseases by increasing ERK1/2 phosphorylation and NF‐κB activation.^22^

In this study, we sought to explore whether USP14 mediates lipid uptake and foam cell formation. Because of the importance of CD36 in the cellular oxLDL uptake and the development of AS, we investigated if CD36 expression is regulated by USP14 and also attempted to study the involved mechanisms in RAW264.7 and THP1 cells.

## 2 METHODS

### 2.1 Materials

IU1 (S7134), obtained from Selleckchem (Houston, TX, USA), was dissolved into DMSO and stored at −20°C. USP14 siRNA (sc‐76817) was from Santa Cruz Biotechnology (Santa Cruz, CA, USA). Anti‐GAPDH (MB001) was purchased from Bioworld Technology (St. Louis Park, MN, USA). Anti‐CD36 (ab133625), anti‐ABCG1 (ab52617), anti‐SR‐B1 (ab217318) and anti‐SR‐A (ab123946) were from Abcam. Anti‐Ubiquitin and anti‐USP14 were obtained from Cell Signaling Technology (MA, USA). Anti‐ABCA1 was from Santa Cruz, and Anti‐Lox‐1 was from R&D system. Oil Red O was purchased from Sigma‐Aldrich. Human oxidized low‐density lipoprotein (oxLDL) (YB‐002) and human Dil‐labelled oxidized low‐density lipoprotein (Dil‐oxLDL) (YB‐0010) were purchased from Yiyuan Biotechnologies (Guangzhou, China). Dynabeads antibody coupling kit was obtained from Life technologies. PMA (P1585) was from Sigma‐Aldrich. Anti‐CD36 (FITC) (ab39022) was purchased from Abcam.

### 2.2 Cell lines and culture conditions

Murine macrophage cell line RAW264.7 and human macrophage cell line THP1 were purchased from ATCC (Manassas, VA, USA). Cells were cultured onto 75 cm^2^ culture flask. RAW264.7 cells were cultured in DMEM (Thermo Fisher Scientific) containing 10% FBS (Biological Industries), and THP1 cells were cultured in RPMI 1640 medium supplemented with 10% FBS.

### 2.3 Analysis of Dil‐oxLDL uptake

The uptake assay was performed using fluorescence‐labelled oxidative LDL (Dil‐oxLDL). RAW264.7 and THP1 cells were plated into confocal cuvette, and THP1 cells were stimulated by PMA (200 ng/mL) for 24 hours. Adherent cells were treated with USP14 inhibitor (IU1) or siRNA for 24 or 48 hours. Dil‐oxLDL (20 μg/mL) was used to incubate with the cells for 6 hours in 37℃. For functional neutralizing assay, before cells were treated with IU1 or USP14 siRNA, cells were pretreated with normal IgG antibody or 10 μg/mL anti‐CD36 antibody for 1 hours. After culture with Dil‐oxLDL, cells were washed with 4 ℃ PBS for three times and fixed with 4% paraformaldehyde for 10 minutes. Then, cells were washed with PBS and DAPI (blue) was used to stain cell nucleus. Fluorescence was tested using fluorescence microscope, and the images were captured. Stained cells were calculated using Image Pro Plus software.

### 2.4 Foam cell formation assay

RAW264.7 and THP1 cells were plated onto slides and treated with USP14 inhibitor or siRNA for indicated time by incubation with oxLDL (50 μg/mL) for 24 hours. Oil Red O was diluted to 0.5% in isopropyl alcohol. Then, the mixture of two parts water and three parts 0.5% Oil Red O was prepared. The treated cells were washed with 4°C PBS thrice and fixed in 4% paraformaldehyde for 10 minutes. PBS was used to wash cells followed by stained with filtered Oil Red O at room temperature for 5 minutes. 60% isopropanol solution into water was used to wash cells, and cells were washed with water for two times. Lastly, cells were stained with haematoxylin for 3 seconds and washed with running water. Images were taken by optical microscope.

### 2.5 Western blot and co‐IP analysis

For Western blot, as we described previously,^23^, ^24^, ^25^ cells were seeded onto 6 cm dishes. After cells’ attachment, cells were exposed to USP14 inhibitor or siRNA and incubated with oxLDL for indicated time. Cell lysates were collected by lysis buffer containing protease inhibitor, PMSF, and then, an equal amount of total protein was separated by SDS‐PAGE and transferred to polyvinylidene difluoride (PVDF) membranes. 5% nonfat mike was used to block the blots on the membranes for 1 hour. Then, the membranes were washed with PBS‐T for 5 minutes thrice. The incubation of primary antibodies was performed overnight and the secondary antibodies were for 1 hour. The reaction between ECL detection reagents and bounded secondary antibodies was performed and exposed to X‐ray films (Kodak, Japan). For co‐IP, antibody was coupled with dynabeads for 16‐24 hours. After then, cell lysates were added. The mixtures containing antibody, lysates and beads were rotated for 1‐2 hours and washed by PBS‐T for three times. Then, the dynabeads were thrown off and the immunocomplexes were suspended with SDS blue loading buffer. The lysis was exposed to 80°C for 10 minutes and then subjected to Western blot.

### 2.6 Flow cytometry assay

To detect the stained cells, cells were treated as the above. RAW264.7 and THP1 cells were exposed to IU1 or USP14 siRNA for the indicated hour and then incubated with Dil‐oxLDL for the additional 6 hours. The treated cells were washed with 4°C PBS thrice and digested with pancreatic enzymes. The washed cells were suspended with PBS and subjected to flow cytometry analysis.

### 2.7 Immunofluorescence assay and intensity analysis

To analyse the intensity of fluorescence, as we previously reported,^26^, ^27^ cells were plated onto chamber slide and exposed to the above. PBS was used to wash the treated cells, and 4% paraformaldehyde was added into slide for 10 minutes. Cells were treated with 0.1% Triton X‐100 (Solarbio Life Science) in PBS for 10 minutes and then blocked with 5% BSA for 30 minutes. Primary antibody diluted with 1% BSA was used to incubate cells overnight and secondary Cy3‐conjugated antibody for 1 hours and fluoroshield mounting medium with DAPI (Abcam) were performed. Images were taken by confocal microscope. Using ImageJ open the file, we firstly convert the file to grey scale 8 and invert. Global calibration and set measurements (area and integrated density) are chosen. Then, we set threshold levels based on the cells in the pictures. The measured results include area and IOD (total intensity). Lastly, we used the mean intensity = IOD/area to compare the effect.

### 2.8 PCR analysis

The assay was performed as we described previously.^28^ RNAs were collected from RAW264.7 treated as the above using the RNeasy Mini Kit (Germany) according to the manufacturer's instructions. The concentration and purity of the purified total RNAs were measured at 260:280 nm. PrimeScript II 1st Strand cDNA Synthesis Kit (TaKaRa Biotechnology) was purchased and used to perform the synthesis of the first‐strand cDNA from an equal amount of RNAs. The mRNA levels of targeted gene were detected using real‐time quantitative PCR. GAPDH was used as an internal control. PCR primers are as follows: CD36: F: 5'‐GAACC TTGAAGGCTTACATCC‐3'; R: 5'‐CCCACACGTGTTGAAC‐3'; β‐actin: F: 5'‐TGGCCGGGACCTGACAGACTA‐3'; R: 5'‐ATCTGAAGCTCGTC CTCTACCGG‐3'.

### 2.9 SiRNA transfection

The assay was performed as previously described.^28^ Briefly, cells were randomly seeded in 6 cm dishes. After 24 hours, 500 μL RPMI opti‐MEM and 5 μL lipofectamine RNA iMax (Invitrogen) reagent become the mixture. Then, the siRNA targeting human USP14 or non‐specific sequences were added into the mixture. Lastly, cells were cultured with the mixture for 48 hours.

### 2.10 Molecular docking

Docking analysis was performed by PatchDock (https://bioinfo3d.cs.tau.ac.il/PatchDock/). The crystal structure of CD36 (PDB ID: http://www.rcsb.org/pdb/search/structidSearch.do?structureId=5LGD) and USP14 (PDB ID: http://www.rcsb.org/pdb/search/structidSearch.do?structureId=6IIM) was obtained from protein data bank (http://www.rcsb.org/). The ligand molecular selection tab was used for upload of pdb files in PatchDock. The geometric shape complementarity score was applied for test of the binding mode and binding affinity. The starting conformation was the best conformations for MD simulation. YASARA was applied for molecular dynamics simulation. All simulations were performed using AMBER 03 force field. Specifically, the protein complex was dissolved with 0.9% NaCl in a dodecahedron box, with a distance of 5 Å between the solute and box. 298 K was considered as the initiation of simulated annealing minimizations, every ten steps lasting for 5 ps with velocities scaling down by 0.9. Following energy minimization, Berendsen thermostat was applied for adjust of temperature of the system to decrease the influence of temperature control. Moreover, every 100 simulation steps, the velocities need to be rescaled, whenever it is converged by the mean of the last 100 detected temperatures. Lastly, a rate of 2 fs was applied to control the 100 ns MD simulations, and every 10 ps for 100 ns MD simulations.

### 2.11 Statistical analysis

At least three times were conducted for every experiment and the representative results are presented. The data were analysed between two groups using unpaired Student's *t* test. The SPSS system was employed. Less than 0.05 of *P* values were considered statistically significant.

## 3 RESULTS

### 3.1 USP14 is a novel CD36‐associated protein in macrophages

**Figure 1:**
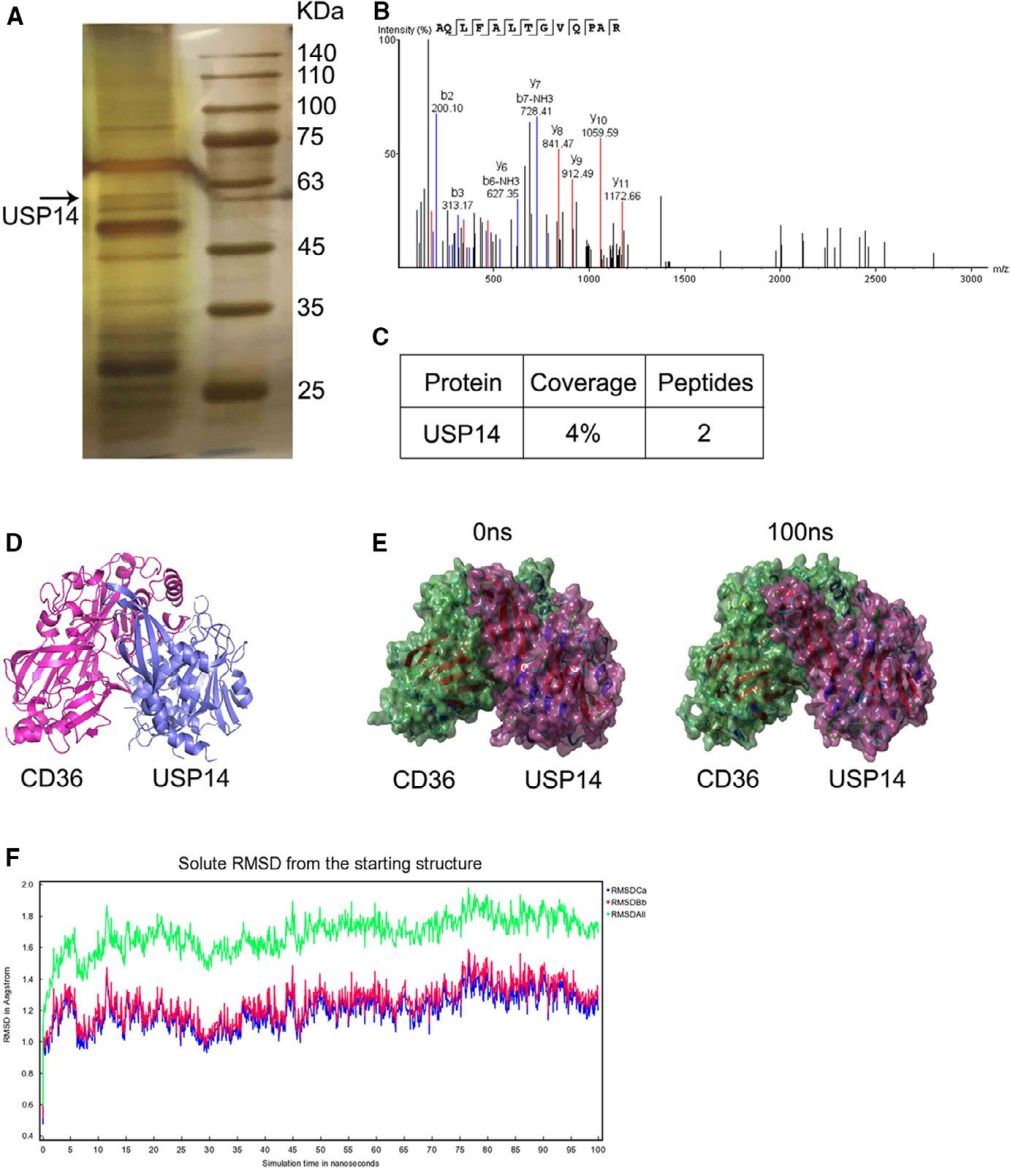
USP14 is a novel CD36‐associated protein in macrophages. (A) Cellular extracts from RAW264.7 cells were immunopurified with anti‐CD36 body beads, followed by SDS‐PAGE and silver staining for mass spectrometry analysis. Representative peptide fragments (B) and coverage (C) of USP14 are shown. (D) Three‐dimensional crystal structure of CD36‐USP14 complex. (E) Surface presentation of the CD36‐USP14 complex crystal structure at 0 ns and 100 ns. (F) Plots of root mean square deviation (RMSD) of C alpha atom (RMSDCa, blue), RMSD of backbone (RMSDBb) and RMSD of all‐heavy atom (RMSDAll)

To better understand the regulation of CD36 in foam cell formation by macrophages, we immunoprecipitated anti‐CD36 antibody for LC‐MS/MS analysis. Firstly, SDS‐PAGE separated the CD36‐associated proteins. We performed silver staining of proteins. The proteins were excised for mass spectrometry. Based on the results, we found that 60‐kD USP14, a deubiquitinating enzyme, was specifically bound to CD36 (Figure 1A‐C). We supposed whether the binding between USP14 and CD36 results from the direct action. To further investigate the interaction of USP14 and CD36, we performed the molecular simulations for these two proteins. As shown in Figure 1D‐F, three‐dimensional crystal structure and CD36‐USP14 complex crystal structure determined that CD36 interacted with USP14. These results indicated that CD36 is associated with the deubiquitinase USP14.

### 3.2 USP14 regulates macrophage expression of scavenger receptor CD36

**Figure 2:**
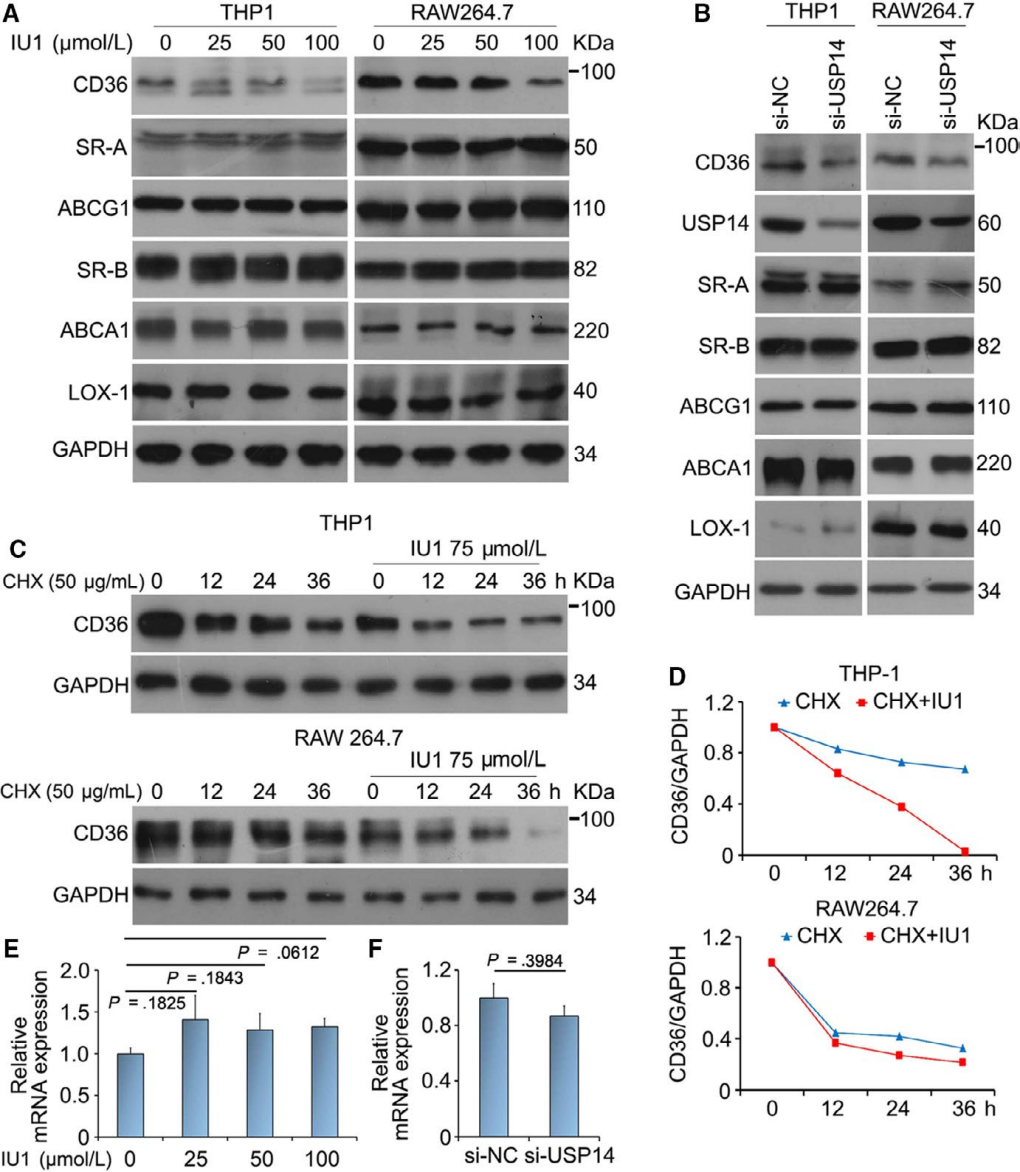
USP14 regulates macrophage expression of scavenger receptor CD36. (A) RAW264.7 and THP1 cells were exposed to DMSO (DM), IU1 (25, 50, 100 μmol/L) for 24 h. Cell lysates were collected followed by Western blot. The proteins levels of CD36, Lox‐1, SR‐A, ABCA1, ABCG1 and SR‐B1 were detected. (B) Cells were treated with either Scramble siRNA or USP14 siRNA. Protein lysates were subjected to Western blot analysis for CD36, SR‐A, Lox‐1, ABCA1, ABCG1 and SR‐B1. (C) The macrophages were exposed to cycloheximide (CHX) for 0, 12, 24, 36 h and co‐treatment of CHX and IU1 at the same time. Western blot assay was used to detect CD36 protein. (D) Quantification of band density was counted. (E, F) Total RNAs were harvested from macrophages treated with IU1 or USP14 siRNA for the indicated time and subjected to RT‐qPCR analysis for CD36 mRNA expression

The formation of foam cell is dependent on scavenger receptors and ABC transporters, including CD36, SR‐A, Lox‐1, ABCA1, ABCB1 and SR‐B1.^29^ To study whether USP14 can influence macrophage scavenger receptors and transporters’ expression, we tested the level of CD36, Lox‐1, SR‐A, ABCA1, ABCG1 and SR‐B1 proteins in THP1 and RAW264.7 cells. The results of Western blot indicated that USP14 inhibitor/siRNA decreased CD36 protein expression in a concentration‐dependent manner. However, the proteins levels of SR‐A, Lox‐1, ABCA1, ABCG1 and SR‐B1 were unchanged by the inhibition of USP14 (Figure 2A, B). Therefore, we think that USP14 regulates the level of CD36 protein rather than that of the others. It is reported that CD36 is a membrane protein and blocking CD36 inhibits lipid uptake and the development of atherosclerosis.^30^ In addition, CD36 is degraded via ubiquitin‐proteasome system (UPS).^31^ Hence, we speculated that deubiquitinase USP14 induced the down‐regulation of CD36 protein by promoting its degradation. Cycloheximide (CHX) was used to treat macrophages. We found that USP14 inhibitor enhanced more rapid decrease in the level of CD36 protein (Figure 2C, D). Except for protein level, we also tested the mRNA level of CD36. The results of RT‐qRCR showed that USP14 deletion did not decrease the mRNA level of CD36 (Figure 2E, F). These findings demonstrated that USP14 inhibition induced the down‐regulation of CD36 in protein rather than in mRNA levels.

### 3.3 OxLDL‐induced up‐regulation of CD36 expression is suppressed by the inhibition of USP14

**Figure 3:**
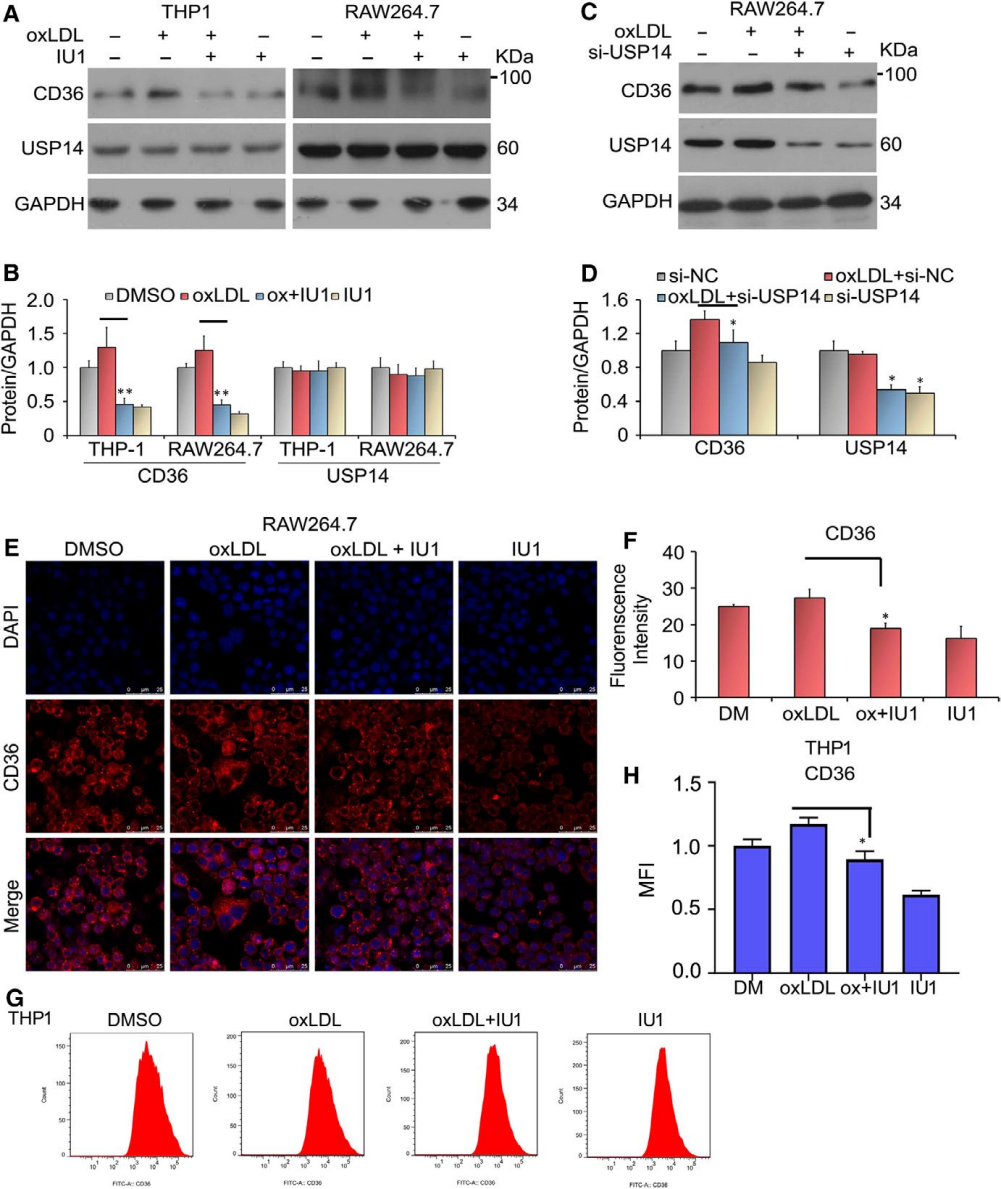
OxLDL‐induced up‐regulation of CD36 expression is suppressed by the inhibition of USP14. (A, C) RAW264.7 or THP1 cells were cultured with IU1/USP14 siRNA or co‐cultured with oxLDL for the indicated time. The expression of CD36 protein was examined using Western blot analysis. (B, D) Quantification of band density was counted. *P < .05, **P < .01 vs. the oxLDL alone‐treated group. (E) RAW264.7 cells post‐IU1 (75 μmol/L) or IU1 + oxLDL (50 μg/mL) treatment for 24 h were fixed and incubated with CD36 antibody. Then, cells were washed and incubated with secondary Cy3‐conjugated antibody. DAPI was used to stain nucleus. Immunofluorescence microscopy showed CD36 (red) and nucleus (blue). (F) The quantity of fluorescence intensity was counted using ImageJ. Three independent experiments were performed. (G) Flow cytometry analysis was applied to measure the expression of CD36 protein. (H) The mean fluorescence intensity was shown. *P < .05 vs. the oxLDL alone‐treated group

Scavenger receptor, CD36, can be activated by oxLDL. Given that USP14 inhibits oxLDL uptake and decreases CD36 expression, we further explored the role of USP14 on CD36 expression when oxLDL is present. We supposed that USP14 has a important role on the expression of CD36 upon macrophages were stimulated by oxLDL. To obtain the purpose, Western blot assay was performed to detect the protein expression of CD36. The result showed that CD36 was increased by the oxLDL treatment. Importantly, inhibition of USP14 down‐regulated the expression of increased CD36 induced by the oxLDL (Figure 3A, B). What is more, the similar results were shown when cells were transfected with USP14 siRNA (Figure 3C, D). As we known, protein expression can be determined by immunofluorescent staining. Therefore, confocal microscopy was used to test the level of CD36 protein. We found that the results of immunofluorescent were similar to that of Western blot (Figure 3E, F). Moreover, we employed the flow cytometry analysis to evaluate the level of CD36 protein. In Figure 3G and H showed that USP14 inhibition decreased the increased CD36 expression by oxLDL‐stimulation. These results suggest that USP14 deletion down‐regulated the expression of CD36 in the presence or absence of oxLDL.

### 3.4 USP14 interacts with and stabilizes CD36 protein

**Figure 4:**
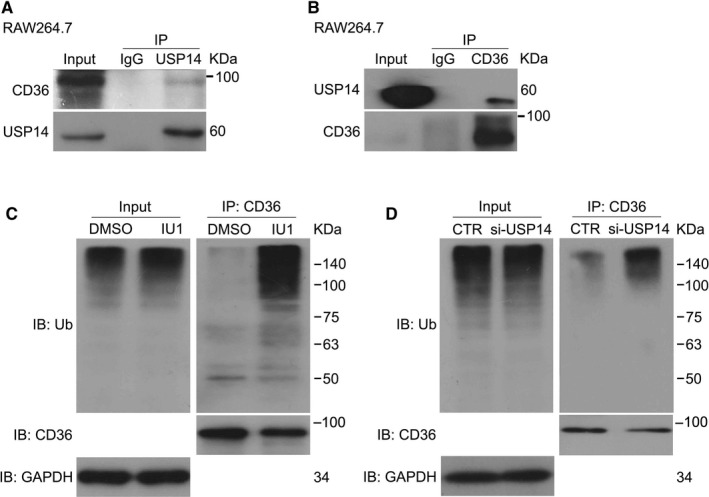
USP14 interacts with and stabilizes CD36 protein. (A) Total proteins were extracted from RAW264.7 cells, immunoprecipitated with USP14 antibody beads and immunoblotted for USP14 and CD36. (B) Then, immunoprecipitated with CD36 antibody beads and immunoblotted for USP14 and CD36. (C, D) RAW264.7 cells were treated with IU1 (75 μmol/L) for 24 h or USP14 siRNA for 48 h. immunoprecipitated with CD36 antibody beads and immunoblotted for ubiquitin (Ub) and CD36. Cells were cultured with MG132 (10 μmol/L) for 6 h before harvest

It has been reported that DUBs regulate their substrate proteins and can directly bind to target protein. To determine whether the interaction exists between USP14 protein and CD36 protein, the co‐immunoprecipitation (co‐IP) assay was performed. The results indicated that USP14 directly binds CD36 protein (Figure 4A, B). We therefore hypothesized that USP14 recruited on the 19S proteasome plays a critical role in the deubiquitination of CD36 and USP14 is a DUB of CD36. We explored the effect of USP14 inhibitor on the abundance of polyubiquitinated CD36. We found that the levels of ubiquitinated CD36 were dramatically increased by the inhibition of USP14 (Figure 4C, D), indicating that USP14 stabilizes CD36 protein via removing the polyubiquitin chains.

### 3.5 Inhibition of USP14 decreases Dil‐oxLDL uptake

**Figure 5:**
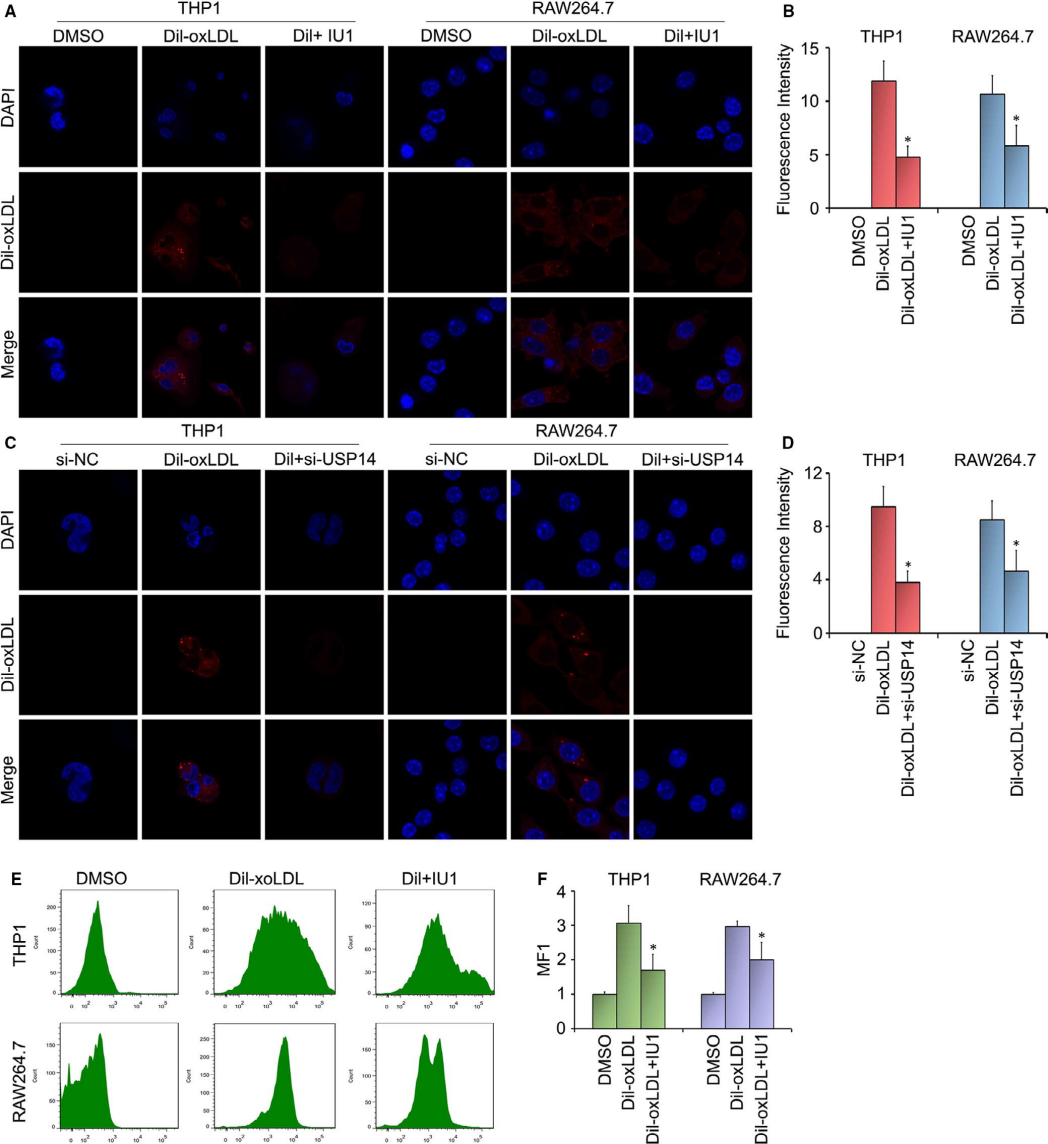
Inhibition of USP14 decreases Dil‐oxLDL uptake. Macrophages were incubated with DMSO, Dil‐oxLDL(20 μg/mL), Dil‐oxLDL + IU1 (75 μmol/L)/USP14 siRNA (50 nmol/L). IU1 for 24 h, Dil‐oxLDL for 6 h and USP14 siRNA for 48 h. The uptake of Dil‐oxLDL was determined by confocal microscopy (A, C) and flow cytometry (E). The experiments were performed at least three times, and the present images were shown. (B, D, F) The quantitative analysis of fluorescence intensity was presented. *P < .05 vs. the oxLDL alone‐treated group

Since the formation of the foam cells is the most important and the initial stage in the development of atherosclerosis, we sought to study the effect of USP14 inhibition on foam cell formation. It is well known that the formation of foam cell results from the oxLDL uptake. Hence, to explore the contribution of USP14 to oxLDL uptake by macrophages, we selected THP1 and RAW264.7 as the target cells. OxLDL is labelled by red fluorescent dye Dil. Confocal microscopy is used to examine the role of USP14 in lipid uptake. We found that the fluorescent intensity was increased in Dil‐oxLDL‐treated cell than that in untreated cells, suggesting Dil‐oxLDL uptake was by macrophages. Significantly, oxLDL uptake was decreased in USP14 inhibitor (IU1) group (Figure 5A). Moreover, USP14 knockdown also induced the inhibition of lipid uptake in THP1 and RAW264.7 cells (Figure 5C). The quantitative analysis of fluorescent intensity demonstrated that USP14 promotes the lipid uptake by macrophages (Figure 5B, D). Flow cytometry results further indicated that USP14 inhibitor/siRNA inhibited cellular oxLDL uptake (Figure 5E, F).

### 3.6 The formation of foam cell is blocked by the inhibition of USP14

**Figure 6:**
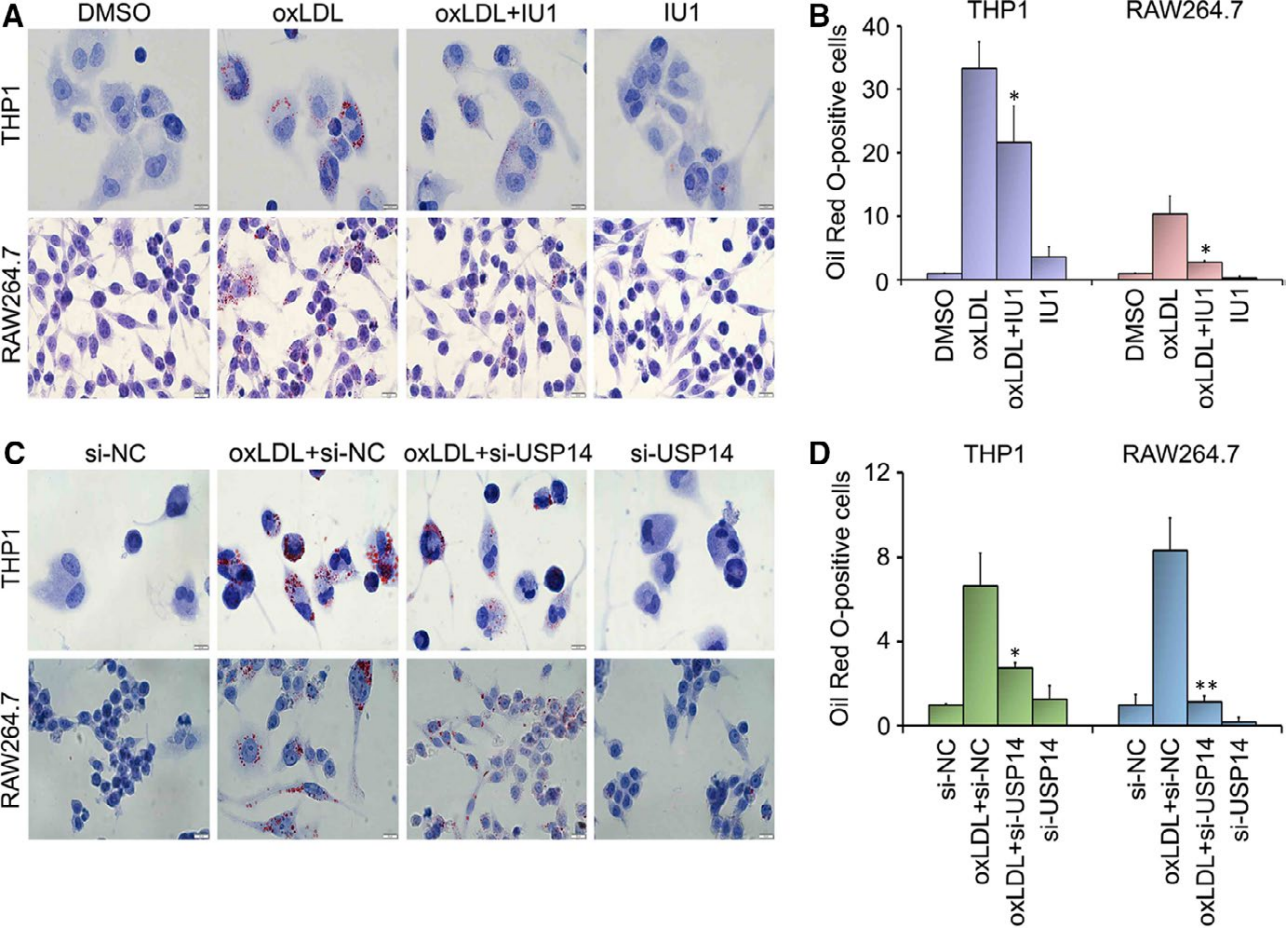
The formation of foam cell is blocked by the inhibition of USP14. (A) RAW264.7 and THP1 cells were treated with DMSO (DM), oxLDL (50 μg/mL), oxLDL (50 μg/mL) +IU1 (75 μmol/L) and IU1 (75 μmol/L) for 24 h. (C) USP14 siRNA replaced IU1 treatment and USP14 siRNA (50 nmol/L) pretreated for 24 h. Then, macrophages were incubated with Scramble siRNA, Scramble + oxLDL (50 μg/mL), USP14 siRNA + oxLDL, and USP14 siRNA for the additional 24 h. Oil Red O was used to stain cells. Representative images are shown from three independent experiments. (B, D) The quantitative analysis of Oil Red O‐positive cells was performed. *P < .05,**P < .01 vs. the oxLDL alone‐treated group

Based on the result that oxLDL uptake was blocked by USP14 inhibitor/siRNA and oxLDL uptake facilitates foam cell formation, we further explored the effect of inhibition of USP14 on the formation of foam cell. To determine this, Oil Red O staining was performed by macrophages after US14 inhibitor treatment. We found that IU1 mediated the decline of formation of foam cell in THP1 and RAW264.7 cells (Figure 6A). Similar results were obtained after the treatment of USP14 knockdown (Figure 6C). The quantitative analysis of cellular accumulated Oil Red O also indicated that decreased foam cell was induced by USP14 inhibition (Figure 6B, D). These results clearly revealed that inhibition of USP14 significantly induced a noticeable decrease of accumulation of cellular lipid droplets.

### 3.7 Inhibition of USP14 decreases Dil‐oxLDL uptake by regulating CD36 expression

**Figure 7:**
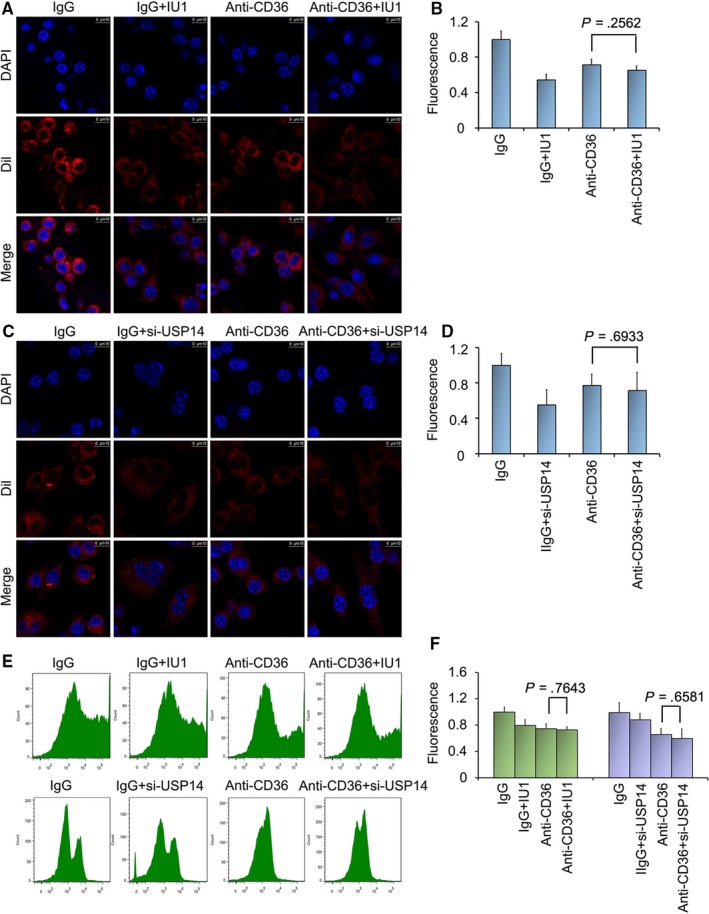
Inhibition of USP14 decreases Dil‐oxLDL uptake by regulating CD36 expression. The macrophages were pretreated with IgG or anti‐CD36 antibody for 1 h, and followed by treated with either Dil‐oxLDL or Dil‐oxLDL + IU1/ USP14 siRNA for the indicated times. (A, C, E) Fluorescence confocal microscopy and flow cytometry were used to analyse total uptake of Dil‐oxLDL. Representative images are shown from three independent experiments. (B, D, F) The quantitative analysis was carried out using Image pro plus

CD36 plays an important role in lipid uptake and contributes to the formation of foam cells. OxLDL uptake is a hallmark of atherosclerosis. To further study the mechanism of USP14 on oxLDL uptake, we have performed the antibody‐dependent blocking assay. The results of confocal microscopy showed that incubation with anti‐CD36 antibody reduced Dil‐oxLDL uptake. Significantly, compared to single anti‐CD36 antibody treatment, adding USP14 inhibitor or siRNA induced no marked difference (Figure 7A‐D). Moreover, the Oil Red O staining was used to detect the effect of USP14 on the formation of foam cell after blocking CD36 antibody. We found that the results in the combination of USP14 inhibitor and anti‐CD36 antibody group were consistent with results in the single treatment group (Figure S1). The results of flow cytometry also indicated that incubation with anti‐CD36 antibody weakened the inhibitory effect of USP14 on the lipid uptake (Figure 7E, F). These findings confirmed that the inhibition of USP14 reduced oxLDL uptake via down‐regulating the expression of CD36.

## 4 DISCUSSION

Foam cells involving in the formation of fatty streak is regarded as be a hallmark of the early stages of atherosclerosis and play an important role in the pathologic development of atherosclerosis.^32^, ^33^, ^34^ The foam cells are characterized by cholesterol esters that exceed 50% of total intracellular cholesterol. Increasing studies have demonstrated that foam cells forming the atherosclerotic reaction are found to be macrophages that are rooted in smooth muscle cells and blood‐borne monocytes.^35^, ^36^ The culture of macrophages is easy, and the cells possess strong phagocytic and adhesive abilities. After the treatment with 50 μg/mL oxLDL for 24 hours, macrophages obtain the typical characteristic of foam cells. Importantly, the formation of foam cell stems from two aspects, including lipid uptake and cholesterol afflux. It is well known that several proteins, such as CD36, Lox‐1 and SR‐A, involve in lipid uptake and ABCA1, ABCG1 and SR‐B1 are for cholesterol transport.^37^

75%‐90% of modified LDL uptake by macrophages are managed by CD36 and SR‐A in vitro. In addition, it was reported that CD36, belonging to scavenger receptor class B family, can bind multiple ligands, such as oxLDL and cell‐derived microparticles.^38^, ^39^ The potential mechanism for regulation of CD36 level has been explored that CD36 is polyubiquitinated via both lysines 48 and 63 of ubiquitin in HEK293 cells or Chinese hamster ovary (CHO) to decrease the expression of CD36 protein.^40^ In addition, fatty acids regulate opposite alteration in CD36 ubiquitination and then modulate CD36 expression and lipid uptake. Therefore, altering CD36 expression may contribution to abnormal oxLDL uptake.

Proteasomes regulate the degradation of proteins. USP14, one of the 19S proteasome‐associated DUBs, dramatically accumulates total K48‐polyubiquitinated proteins, indicating that USP14 mediated the degradation of most proteins.^41^, ^42^ Our previous studies have shown that USP14 stabilizes AR protein expression by removing the K48 ubiquitin chain on AR in prostate cancer cells and breast cancer cells. Moreover, except for in cancer cells, we also found that USP14 regulates the progression of cardiac hypertrophy via promoting GSK‐3β phosphorylation and USP14 mediates LPS‐induced inflammation by activating ERK1/2 and NF‐κB pathways.^22^, ^43^ However, the function of USP14 in the development of atherosclerosis remains unknown.

In the present study, we have demonstrated that inhibition of USP14 attenuated oxLDL uptake in RAW264.7 and THP1 macrophages for the first time. Significantly, USP14 inhibition or knockdown decreased oxLDL‐induced the formation of foam cell by macrophages. Based on the results and considering the necessity of scavenger receptors in the mechanism of foam cell formation, we explored the correlation between USP14 and CD36. Our study showed that the expression of CD36 protein was decreased by inhibition of USP14. Additionally, USP14 does not affect markedly the expressions of SR‐A and Lox‐1 proteins and the mRNA level of CD36. Moreover, ABCA1, ABCG1 and SR‐B1 play key roles in cholesterol transport which is responsible for the formation of foam cell. Hence, we also tested whether USP14 has a main effect on cholesterol efflux. The results showed that USP14 inhibition has no significant effect on the level of mediator of cholesterol efflux. Therefore, we think that USP14 mediates the formation of foam cell by mainly regulating the expression of CD36. When oxLDL is present, the expression of CD36 is up‐regulated. Importantly, USP14 inhibition or knockdown blocked the up‐regulation of CD36 induced by oxLDL.

Subsequently, we further demonstrated the molecular mechanism by which USP14 stabilizes the expression of CD36 protein. We found that USP14 interacts with CD36 and identified that USP14 as an CD36 DUB by cleaving its polyubiquitin chains in macrophages to inhibit proteasome‐mediated degradation of CD36. To investigate the role of CD36 in which the lipid uptake is reduced by the inhibition of USP14, we blocked the expression of CD36. When the CD36 is inactivated, USP14 does not noticeably influence on the inhibition of oxLDL uptake. Data presented in this study revealed that the inhibition of USP14 reduces the formation of foam cell by mainly promoting the degradation of CD36 (Figure S2).

In conclusion, our findings show that USP14 inhibition suppresses oxLDL uptake, which may mainly be due to the down‐regulation of CD36 level, and has no effect on cholesterol efflux. These effects result in the decrease on the formation of foam cell by macrophages. Atherosclerosis is the core cause of death worldwide, and the underlying molecular mechanism still be worthy to further research. Our study describes a possible mechanism how USP14 participates in the pathologic processes of atherosclerosis and provides a new target for clinical therapy in atherosclerosis.

## CONFLICT OF INTEREST

The authors declare that they have no conflict of interest.

## AUTHOR CONTRIBUTIONS

NL, and SL designed the experiments. FZH, XX, RC, RX, QX, ML, XC and BL performed the experiments. NL, SL, FZH and XX wrote the manuscript. All authors read and approved the final manuscript.

## Data Availability

The data that support the findings of this study are available from the corresponding author upon reasonable request.
