# Supplementary material for: Inhibition of USP14 suppresses the formation of foam cell by promoting CD36 degradation
Source: J Cell Mol Med. 2020 Jan 22;24(6):3292–302. doi: 10.1111/jcmm.15002 (PMC7131911; doi:10.1111/jcmm.15002)
Supplement: Supplementary file 1 [file JCMM-24-3292-s001.docx]

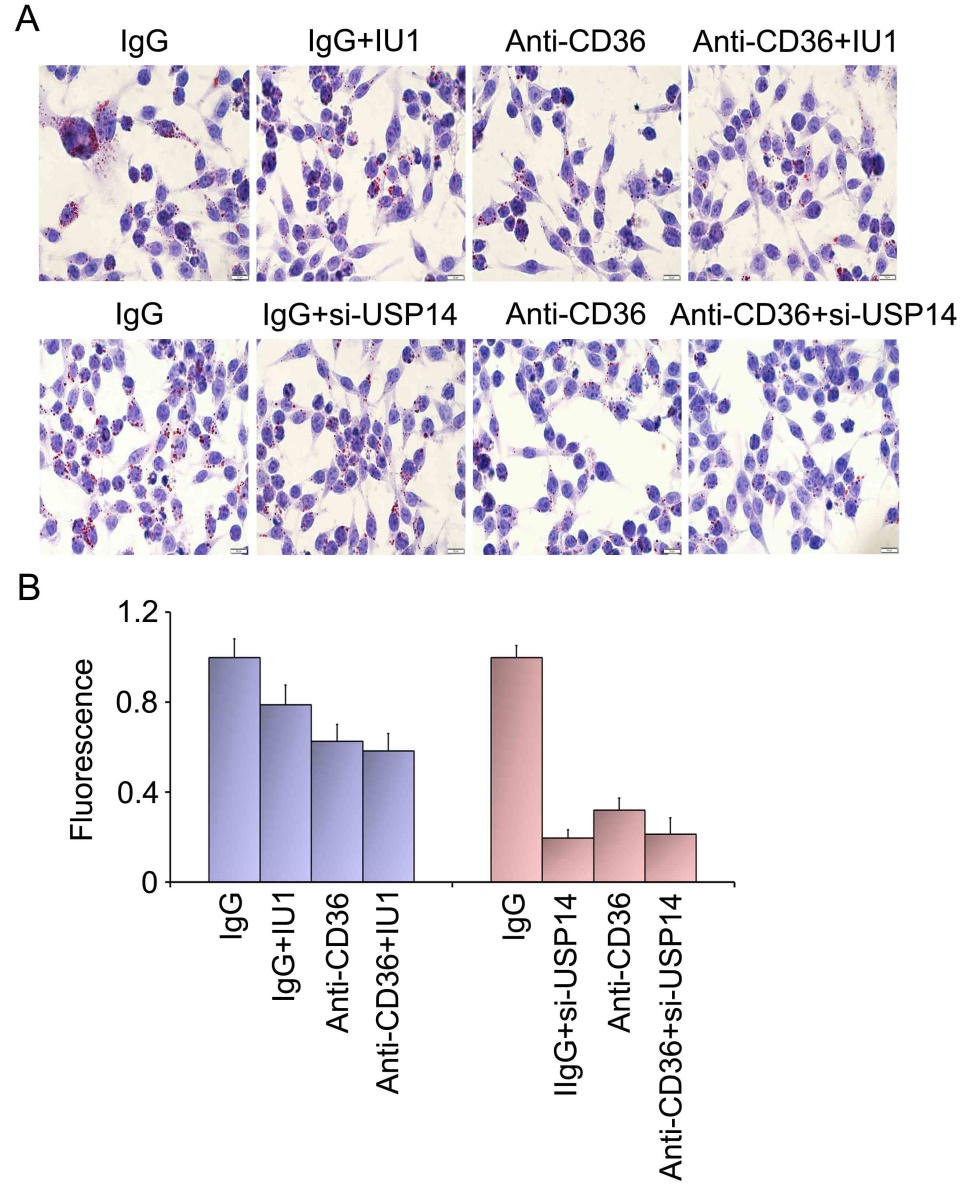


**Fig. S1 USP14 inhibition induced-decreased lipid uptake depends on CD36 expression.** (A) RAW264.7 cells were pretreated with IgG or anti-CD36 antibody for 1 hour, and followed by treated with either oxLDL or oxLDL + IU1/ USP14 siRNA for the indicated times. The treated cells were stained with Oil Red O. Representative images are shown from three independent experiments. (B) The quantitative analysis of Oil Red O-positive cells were performed.


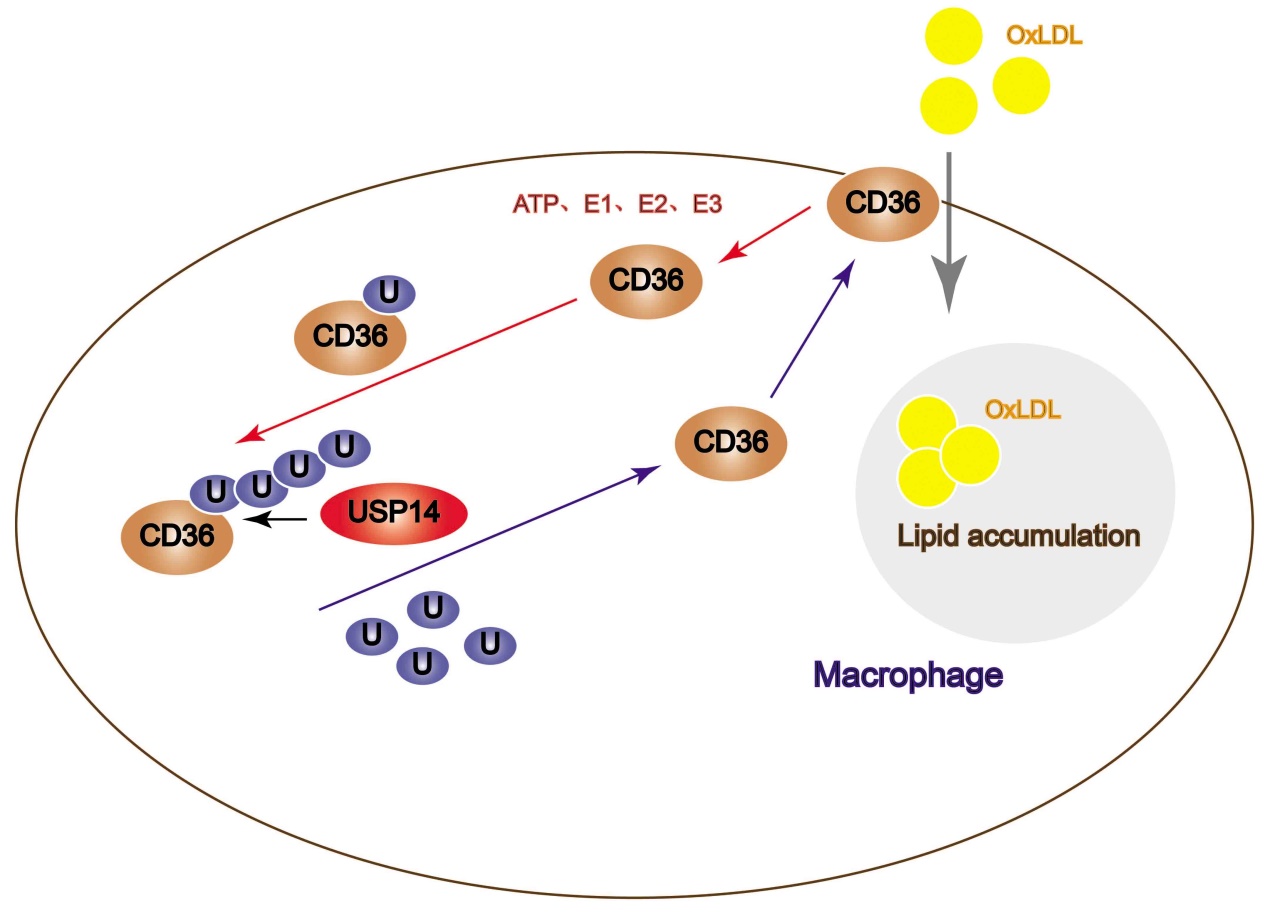


**Fig. S2 A proposed model of regulation of USP14 on the formation of foam cell via cleaving the ubiquition on CD36.**
